# Skeletal muscle fibre type-dependent effects of atorvastatin on the PI3K/Akt/mTOR signalling pathway and atrophy-related genes in rats

**DOI:** 10.1007/s11033-024-10005-w

**Published:** 2024-10-17

**Authors:** Anna Gawedzka, Malgorzata Knapik-Czajka, Jagoda Drag, Malgorzata Belczyk, Edyta Radwanska, Dariusz Adamek

**Affiliations:** 1 Department of Biochemical Analytics, Faculty of Pharmacy, Jagiellonian University Medical College, Medyczna 9 St., Krakow, 30-688 Poland; 2 Department of Neuropathology, Faculty of Medicine, Jagiellonian University Medical College, Krakow, Poland

**Keywords:** Akt pathway, Atrophy-related genes, Atorvastatin, Skeletal muscle fibres

## Abstract

**Background:**

One of the probable causes of statin myotoxicity is an imbalance between protein synthesis and degradation. These processes are regulated by the PI3K/Akt/mTOR pathway and the ubiquitin‒proteasome system (UPS). The aim of this study was to assess whether the effects of atorvastatin on PI3K/Akt/mTOR pathway downstream proteins, the FoxO3a transcription factor and the UPS genes, i.e., MuRF-1 and MAFbx, depend on muscle fibre type.

**Methods and results:**

Atorvastatin (50 mg/kg) was administered to Wistar rats. The levels of selected PI3K/Akt/mTOR pathway proteins were assayed via Western blotting, whereas MuRF-1, MAFbx and FoxO3a mRNA levels were measured using reverse transcription quantitative polymerase chain reaction (RT‒qPCR). Gomöri trichrome staining was performed to assess skeletal muscle pathology. A decrease in the P-Akt/Akt ratio was observed in the gastrocnemius muscle (MG), whereas an increase in the P-Akt/Akt ratio was observed in the soleus muscle (SOL). FoxO3a gene expression increased in the SOL and extensor digitorum longus (EDL) muscles. MuRF-1 gene expression increased in the MG, and MAFbx expression increased in the EDL. No histopathological changes were observed in any of the tested muscles.

**Conclusions:**

In the absence of overt muscle damage, atorvastatin decreased the P-Akt/Akt ratio in the MG, indicating an increase in inactive Akt. Consistent with the decrease in Akt activation, rpS6 phosphorylation decreased. In SOL, atorvastatin increased the P-Akt/Akt ratio, indicating Akt activation. P-FoxO3a and the P-FoxO3a/FoxO3a ratio increased, suggesting that FoxO3a inactivation occurred. Moreover, in the SOL, atorvastatin did not affect the expression of atrophy-related genes. These findings indicate that atorvastatin has no adverse effect on the Akt pathway in the SOL. Our results showed that the effects of atorvastatin on the Akt signalling pathway and atrophy-related gene expression depend on muscle type.

**Supplementary Information:**

The online version contains supplementary material available at 10.1007/s11033-024-10005-w.

## Introduction

Statins, 3-hydroxy-3-methylglutaryl-CoA (HMG-CoA) reductase inhibitors, are commonly used as lipid-lowering drugs. They are the most effective and well-tolerated oral drugs for the prevention and treatment of cardiovascular diseases resulting from dyslipidaemia [1]. The most often prescribed medication in this class is atorvastatin [2]. It is highly effective at even low doses, giving it an advantage over the other statins [3].

Statins appear to be safe and have relatively few side effects and affect mainly skeletal muscle. Given its lipophilic character, atorvastatin is more likely to cause muscular events than are hydrophilic statins [4]. There are several possible mechanisms that may explain the side effects of statins on skeletal muscle, such as PI3K/Akt/mTOR pathway disruption [5, 6] and the induction of muscle protein degradation due to increased expression of atrophy-related genes [7].

The serine/threonine kinase protein kinase B (Akt), which is one of the main transducers of the PI3K/Akt/mTOR signalling pathway, is involved in many cellular processes, including protein metabolism [8]. Activated PI3K phosphorylates and activates Akt and thus regulates protein synthesis via mammalian target of rapamycin kinase (mTORC) and glycogen synthase kinase 3β (GSK3β) [9]. The activation of mTORC1 by Akt results in the phosphorylation of eukaryotic initiation factor 4E-binding protein 1 (4E-BP1), which leads to the release of eIF4E, making it available for translation. In addition, mTORC1 phosphorylation of ribosomal protein S6 kinase 1 (S6K1) leads to the phosphorylation and activation of ribosomal protein S6 (rpS6), a crucial structural component of the 40 S subunit of the eukaryotic ribosome [10]. Therefore, phosphorylated rpS6 (P-rpS6) stimulates protein synthesis [11].

Moreover, Akt inhibits protein degradation via phosphorylation and thus inactivation of Forkhead box O family transcription factors (FoxOs). Among all FoxO factors, FoxO3a plays a pivotal role in the ubiquitin‒proteasome system (UPS) in skeletal muscle [12]. FoxO3a regulates the gene expression of RING finger protein-1 (MuRF-1) and atrogin-1, also called muscle atrophy F-box (MAFbx) [13, 14]. Both the MuRF-1 and MAFbx genes, referred to muscle atrophy-related genes, encode E3 ligases that take part in the ubiquitination of numerous skeletal muscle proteins and target them for degradation in proteasomes [12]. MuRF-1 and MAFbx gene overexpression is associated with a shift in protein balance from net synthesis to net degradation [13].

Rat hindlimb skeletal muscles differ in the composition of muscle fibres. The soleus muscle (SOL) contains mainly type I, slow-twitch oxidative fibres, whereas the extensor digitorum longus (EDL) is composed mainly of type II, fast-twitch, glycolytic fibres [15]. The gastrocnemius muscle (MG) is heterogeneous in terms of its fibre-type proportion [16, 17] and contains both type I and type II fibres. Histopathological studies have shown that statins cause necrotic lesions mainly in type II fibres, i.e., fast-twitch, glycolytic muscle fibres, and to a lesser extent, in type I fibres, i.e., slow-twitch, oxidative muscle fibres [18, 19]. However, the exact molecular mechanism of these changes remains unclear. We hypothesize that atorvastatin distinctly modifies the PI3K/Akt/mTOR pathway and thus affects different proteins that participate in protein turnover, including rpS6 and transcription factors. Therefore, we investigated the effect of atorvastatin on the PI3K/Akt/mTOR pathway in muscles composed of different types of fibres.

The principal purpose of our study was to compare the effects of short-term atorvastatin administration on the protein levels of total Akt and its active form, phospho-Akt (P-Akt), in the SOL, EDL and MG. Moreover, the protein levels of downstream targets of the PI3K/Akt/mTOR pathway, i.e., rpS6, phospho-rpS6 (P-rpS6), FoxO3a and phospho-FoxO3a (P-FoxO3a), were assessed. Additionally, this study aimed to assess whether atorvastatin affects the expression of E3 ligase genes, i.e., MuRF-1 and MAFbx, and the FoxO3a transcription factor gene. We also wanted to determine whether atorvastatin affects skeletal muscle structure; thus, we decided to examine the EDL, which contains predominantly glycolytic, type II fibres that are the most sensitive to necrotic changes occurring under statin treatment.

## Materials and methods

### Animals

Sixteen male Wistar rats from an outbred stock (6–7 weeks; 165.8 ± 1.3 g) were obtained from the breeding facility of Jagiellonian University, Faculty of Pharmacy. The animals were maintained in standard laboratory cages (two per cage) under controlled conditions, including an artificial 12:12 h light/dark cycle, temperature of 22 ± 2 °C, humidity levels of 50 ± 10% and ad libitum access to standard chow and tap water. During the experimental period, the weight and food consumption of the rats were monitored daily.

All experimental procedures followed the International Guiding Principles for Biomedical Research Involving Animals, the European Union Guidelines, and the Polish Law on the Protection of Animals, and were approved by the First Local Ethics Committee on Animal Research in Krakow, Poland (Permission Number: 64/2018).

### Experimental design

**Fig. 1:**
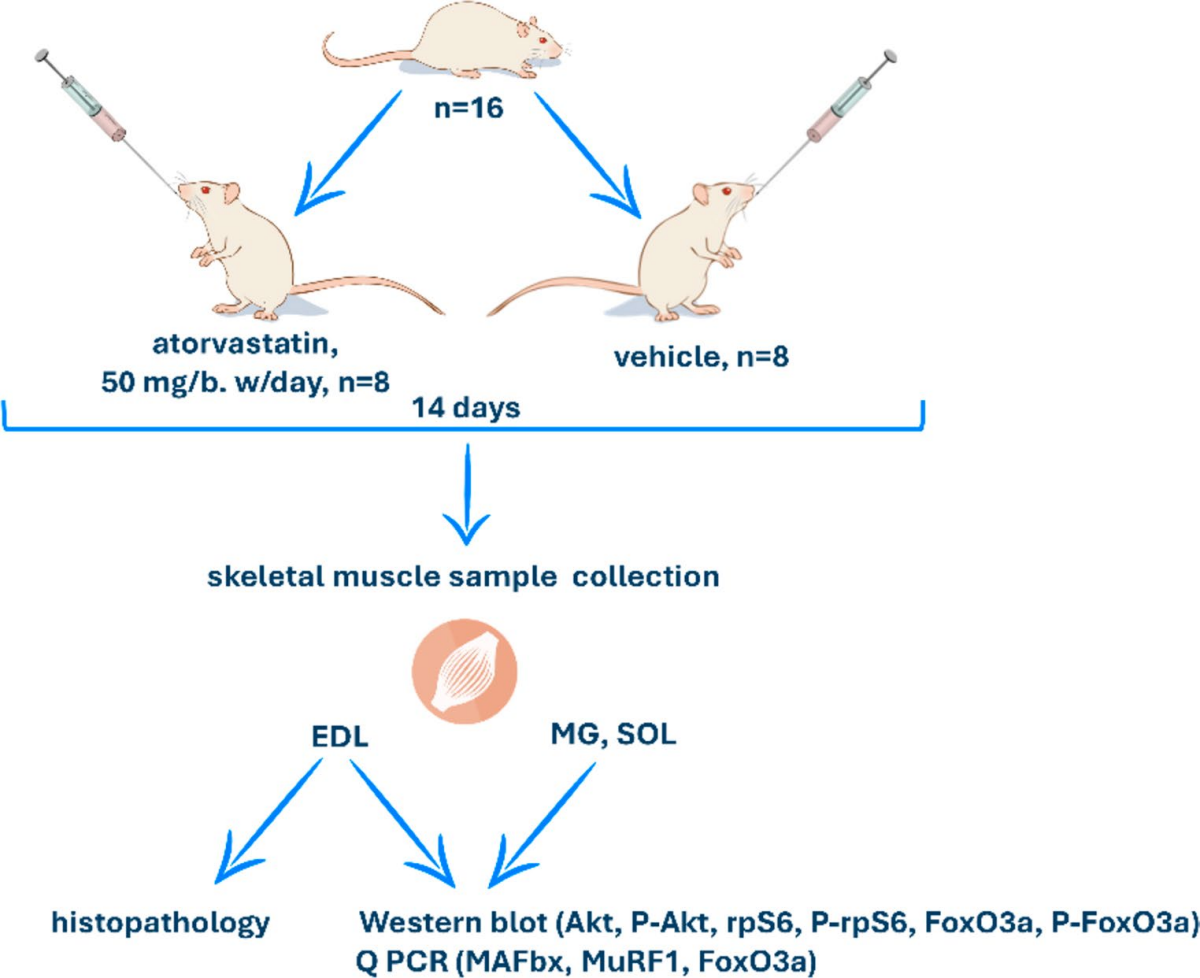
Experimental design; EDL- extensor digitorum longus; MG- gastrocnemius muscle; SOL- soleus muscle

The rats were randomly divided into 2 groups: the atorvastatin (Atorv) group (*n* = 8) and the control (Ctl) group (*n* = 8). The rats received 50 mg/kg/day atorvastatin calcium salt trihydrate (TCI- Tokyo Chemical Industry, Japan) in 0.5% methylcellulose solution (vehicle) for 14 days. The dose of atorvastatin was 1% of the LD_50_ and was chosen on the basis of the methods of Simsek Ozek et al. [20] and Elshama et al. [21]. Ctl group rats were treated for 14 days only with vehicle. Both atorvastatin and vehicle were administered via oral gavage at a volume of ∼ 0.5 ml. On Day 15, 24 h. following the last atorvastatin or vehicle application, the rats were sacrificed by decapitation, and blood and MG, SOL and EDL muscles were rapidly collected and subjected to various analyses, as described below (Fig. 1).

### Serum creatine kinase measurement

Serum creatine kinase (CK) levels were measured using a UV‒VIS Cary 100 spectrophotometer (Varian Medical Systems, Australia) and a CK Kit (BioMaxima, Poland) following the protocol provided by manufacturer.

### Protein extraction and Western blotting

Approximately 50 mg of appropriate muscle (SOL, EDL or superficial white portion of MG) was weighed. Total protein extraction was performed according to the procedure described previously [22, 23].

Equal amounts (30 µg) of total protein were loaded onto 12% SDS‒polyacrylamide gels and separated. Semi-quantitative protein analysis was performed via Western blotting according to the procedure previously published [22, 23]. Membranes were incubated overnight at 4 °C with the appropriate primary antibodies (1:1000 dilution) against total Akt (Cat. No. 4691), phospho-Akt (Ser 473, Cat. No. 4060), total rpS6 (Cat. No. 2217), phospho-rpS6 (Ser235/236 Cat. No. 4858) (Cell Signaling Technology, Danvers, MA, USA), total FoxO3a (Cat. No. MA514932), phospho-FoxO3a (Ser253, Cat. No. PA537578) (Invitrogen, Thermo Fisher Scientific, Waltham, MA, USA). Glyceraldehyde 3-phosphate dehydrogenase (GAPDH) (Cat. No. 2118) (Cell Signaling Technology, Danvers, MA, USA) served as a reference protein. After incubation with primary antibodies, the membranes were incubated with goat anti-rabbit IgG-H&L (HRP) (Cat. No. ab6721; Abcam, UK) secondary antibody using a 1:7000 dilution. The protein bands were visualized via an enhanced chemiluminescence method (Clarity Western ECL Substrate Bio-Rad, CA, USA) with a GeneSys G: BOX Chemi-XR5 gel doc system (Syngene, UK). The values of optical density determined for each protein were normalized to the optical density of GAPDH and are presented as the relative level (protein of interest/GAPDH).

### RNA isolation and reverse-transcription quantitative PCR

Approximately 50 mg of appropriate muscle (MG, SOL or EDL) was weighed, ground via a nitrogen-cooled mortar and pestle, and homogenized in RNA Extracol (EURx, Poland), after which total RNA was extracted according to the manufacturer’s protocol. RNA concentration and quality were measured via a UV‒VIS Cary 100 spectrophotometer (Varian Medical Systems, Australia). Reverse transcription was performed with a High Capacity cDNA Reverse Transcription Kit (Thermo Fisher Scientific, Waltham, MA, USA). qPCR was performed using TaqMan Fast Advanced Master Mix (Thermo Fisher Scientific, Waltham, MA, USA) and appropriate TaqMan probes (*MuRF-1*, Rn00590197_m1; *MAFbx1*, Rn00591730_m1; *FoxO3a*, Rn01441087_m1). The expression of the reference gene, GAPDH (Rn99999916_s1), was quantified to control for variation in cDNA amounts. The qPCR tests were performed using a StepOnePlus Real-Time PCR System instrument (Applied Biosystems, CA, USA). Approximately 25 ng of cDNA was used for each reaction and prepared in triplicate. The relative expression was calculated as 2 ^(-ΔΔCt)^, where ΔΔCt ^(Ct gene - Ct GAPDH)^.

### Histopathological studies

Gomöri trichrome (GT) staining was performed to assess possible skeletal muscle pathology. The EDL muscle samples were frozen, and serial cross-sections were cut via a Leica CM 1850 UV cryostat and placed on microscope slides. Then, the slides were incubated in Harris haematoxylin solution (Thermo Fisher Scientific, Waltham, MA, USA) for 3 min, washed in running water for 5 min, rinsed in distilled water, incubated with modified Gomöri solution (Chromotrope 2R, Fast Green FCF, phosphotungstic acid, glacial acetic acid, distilled water) at 37 °C for 30 min and washed with 0.2% glacial acetic acid solution. The stained tissue samples were dehydrated twice with 100% ethanol for 5 min and cleared with xylene. The samples were mounted using Cytoseal mounting medium (Fisher Scientific, Waltham, MA, USA). Images were captured with a Nikon Eclipse 80i microscope.

### Data analysis

To assess the proportions of the active or inactive form of the protein (depending on the type of protein), the ratio of the phosphorylated form to the corresponding total protein (phospho-protein of interest/total protein of interest) was calculated.

### Statistical analysis

The data are presented as the means ± standard errors of the means (means ± SEMs) from two independent experiments.

The statistical analyses were performed using the statistical package STATISTICA 13.3 (Stat Soft, Tulsa, OK, USA) and GraphPad Prism 8.0 (GraphPad Software, San Diego, CA, USA). Differences among groups were determined via the Mann‒Whitney U test and t test. The level of statistical significance was set at *p* < 0.05.

## Results

### Atorvastatin reduces food intake and body weight

We observed a significant decrease (*p* < 0.01, Table 1) in food intake in the Atorv group compared with the Ctl group. Moreover, rats in the Atorv group presented a decrease (*p* < 0.05) in body weight compared with those in the Ctl group. However, the decrease in body weight observed in the Atorv group was not accompanied by a change in the wet mass of MG (*p* > 0.05).

There was no difference in the serum CK activity between the Ctl group and the Atorv group (11347 ± 525 U/L and 16463 ± 2186 U/L, respectively; *p* > 0.05).

**Table 1:** Body weight, food intake and gastrocnemius muscle weight

Variables	Ctl, *n* = 8	Atorv, *n* = 8
Final body weight, g	268.2 ± 3.2	236.8 ± 12.99*
Food intake, g/day ^#^	153.1 ± 2.2	126.7 ± 4.7**
MG wet mass, g	2.2 ± 0.15	2.1 ± 0.29
MG/body weight, %	0.8 ± 0.06	0.9 ± 0.1

Data are presented as means ± SEMs. Abbreviations: Ctl, control group, Atorv, atorvastatin treated group; MG, gastrocnemius muscle, ^#^ 2 rats per cage; *p < 0.05; **p < 0.01

### Atorvastatin reduces the levels of phosphorylated Akt and rpS6 but not FoxO3a in rat MG

**Fig. 2:**
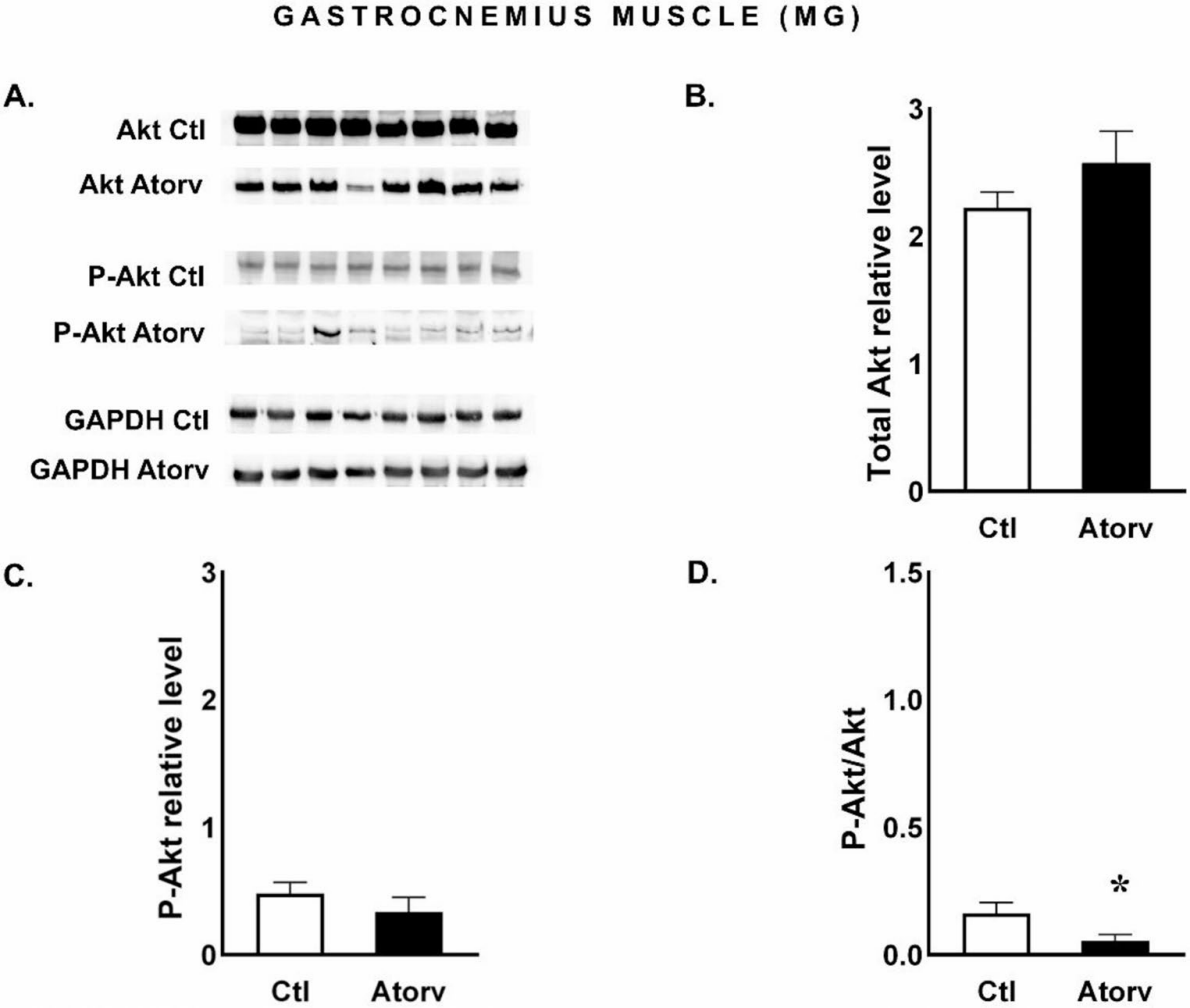
Semi-quantitative analysis of atorvastatin effects on Akt and P-Akt protein levels and the P-Akt/Akt ratio in the MG performed via Western blotting. **A**: Representative immunoblots for Akt, P-Akt and GAPDH; **B**: Relative Akt protein levels; **C**: Relative P-Akt protein levels; **D**: P-Akt/Akt ratio. *Abbreviations* Ctl, control group; Atorv, atorvastatin-treated group. The data are presented as the means ± SEMs of *n* = 8 animals per group. *p < *0.05*

Our findings indicated that atorvastatin did not affect (*p* > 0.05) the total protein levels of Akt or P-Akt (Fig. 2B and C). However, the ratio of P-Akt to total Akt protein (P-Akt/Akt) was significantly lower (*p* < 0.05) in the Atorv group than in the Ctl group (Fig. 2D), indicating a lower level of the active, phosphorylated form of Akt.

**Fig. 3:**
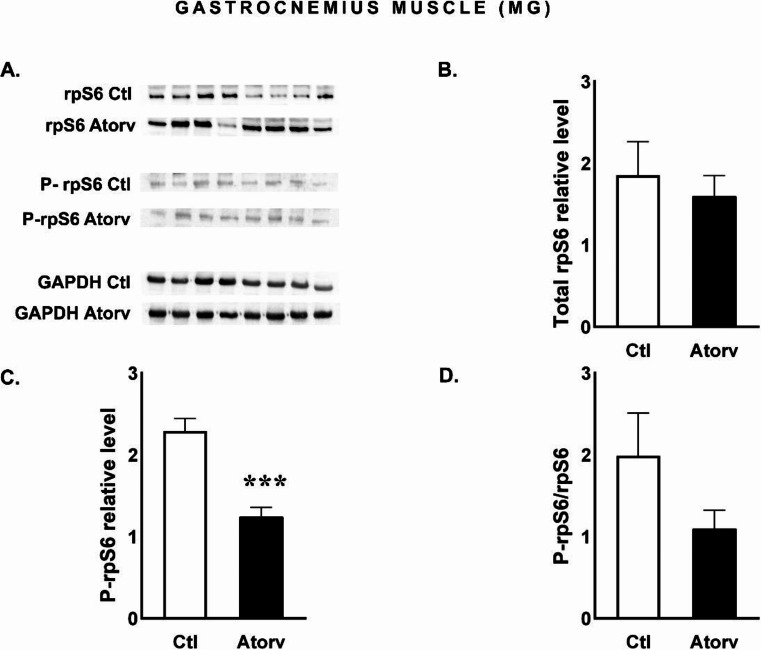
Semi-quantitative analysis of atorvastatin effects on rpS6 and P-rpS6 protein levels and the P-rpS6/rpS6 ratio in the MG performed via Western blotting. **A**: Representative immunoblots for rpS6, P-rpS6 and GAPDH; **B**: Relative rpS6 protein levels; **C**: Relative P-rpS6 protein levels; **D**: P-rpS6/rpS6 ratio. *Abbreviations* Ctl, control group; Atorv, atorvastatin-treated group. The data are presented as the means ± SEMs of *n* = 8 animals per group. ***p < 0.001

The level of total rpS6, a downstream target of Akt, did not change (*p* > 0.05) after atorvastatin treatment. However, in the Atorv group, the level of active, phosphorylated rpS6 protein decreased significantly (*p* < 0.05) compared with that in the Ctl group, but the P-rpS6/rpS6 ratio did not change (Fig. 3B-D).

**Fig. 4:**
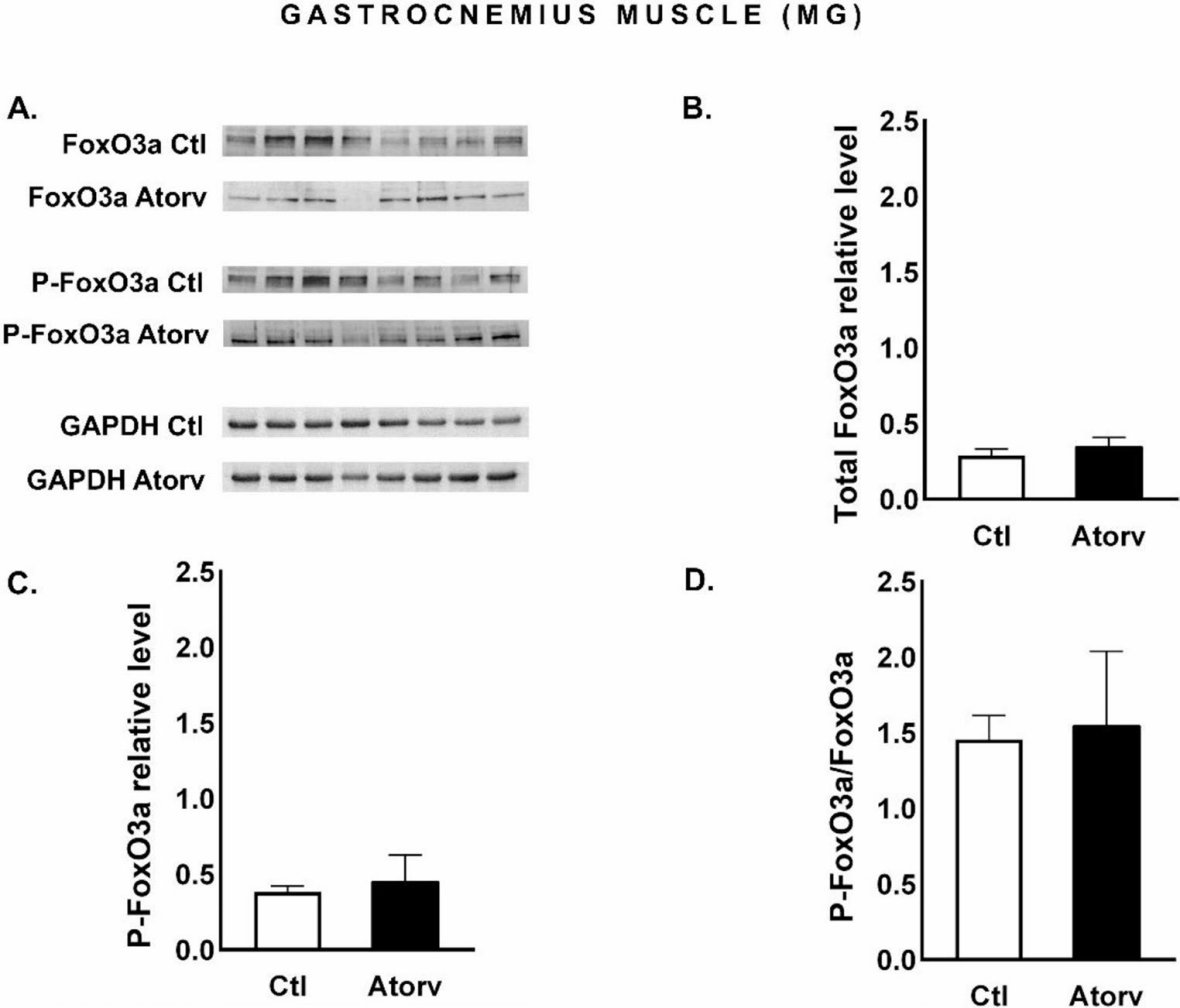
Semi-quantitative analysis of atorvastatin effects on FoxO3a and P-FoxO3a protein levels and the P-FoxO3a/FoxO3a ratio in the MG performed via Western blotting. **A**: Representative immunoblots for FoxO3a, P-FoxO3a and GAPDH; **B**: Relative FoxO3a protein levels; **C**: Relative P-FoxO3a protein levels; **D**: P-FoxO3a/FoxO3a ratio. *Abbreviations* Ctl, control group; Atorv, atorvastatin-treated group. The data are presented as the means ± SEMs of *n* = 8 animals per group

Unlike those of Akt and rpS6, the FoxO3a phosphorylation status remained unaffected by atorvastatin treatment. Atorvastatin did not affect total FoxO3a or P-FoxO3a levels or the P-FoxO3a/FoxO3a ratio (*p* > 0.05, Fig. 4B-D).

### Atorvastatin influences the phosphorylation of Akt and FoxO3a but not rpS6 in the rat SOL

**Fig. 5:**
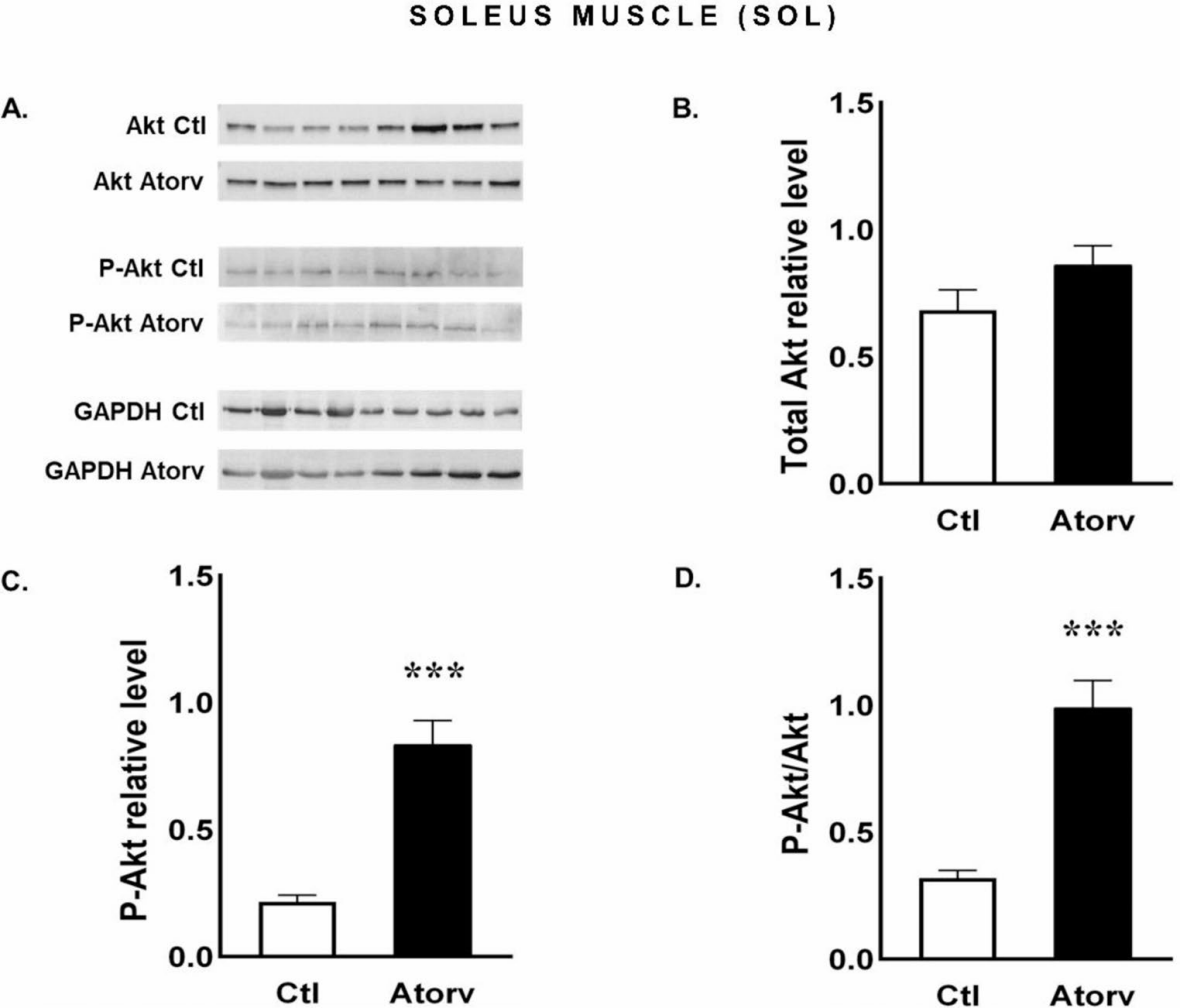
Semi-quantitative analysis of atorvastatin effects on Akt and P-Akt protein levels and the P-Akt/Akt ratio in the SOL muscle performed via Western blotting. **A**: Representative immunoblots for Akt, P-Akt and GAPDH; **B**: Relative Akt protein levels; **C**: Relative P-Akt protein levels; **D**: P-Akt/Akt ratio. *Abbreviations* Ctl, control group; Atorv, atorvastatin-treated group. The data are presented as the means ± SEMs of *n* = 8 animals per group. ***p < 0.001

The Western blot results revealed an increase (*p* < 0.05) in P-Akt levels in the SOL muscle (Fig. 5C), whereas the total Akt protein level did not change (*p* > 0.05) in the Atorv group compared with the Ctl group (Fig. 5B). Therefore, a significantly greater (*p* < 0.05) P-Akt/Akt ratio was detected in the Atorv group than in the Ctl group (Fig. 5D).

**Fig. 6:**
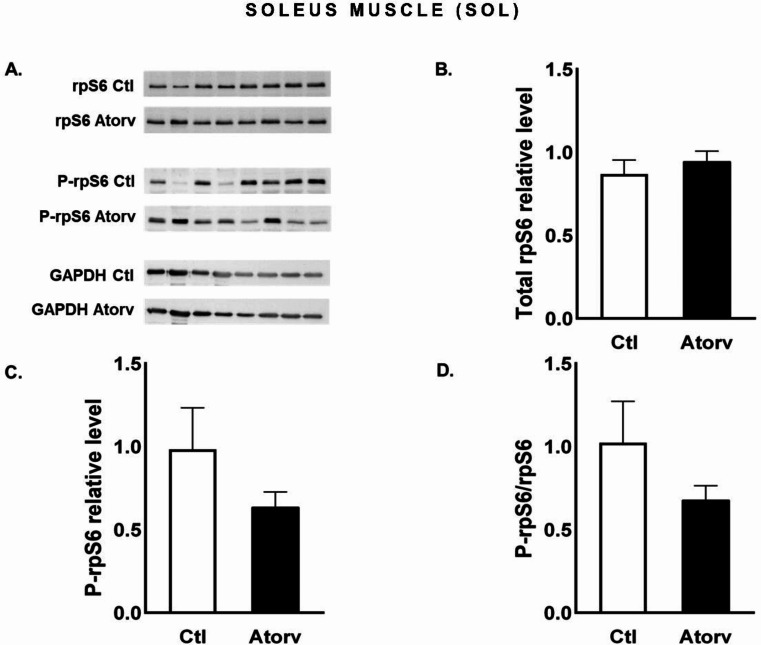
Semi-quantitative analysis of atorvastatin effects on rpS6 and P-rpS6 protein levels and the P-rpS6/rpS6 ratio in the SOL muscle performed via Western blotting. **A**: Representative immunoblots for rpS6, P-rpS6 and GAPDH; **B**: Relative rpS6 protein levels; **C**: Relative P-rpS6 protein levels; **D**: P-rpS6/rpS6 ratio. *Abbreviations* Ctl, control group; Atorv, atorvastatin-treated group. The data are presented as the means ± SEMs of *n* = 8 animals per group

In the SOL muscle, atorvastatin did not affect (*p* > 0.05) P-rpS6 or total rpS6 protein levels or the P-rpS6/rpS6 ratio (Fig. 6B-D).

**Fig. 7:**
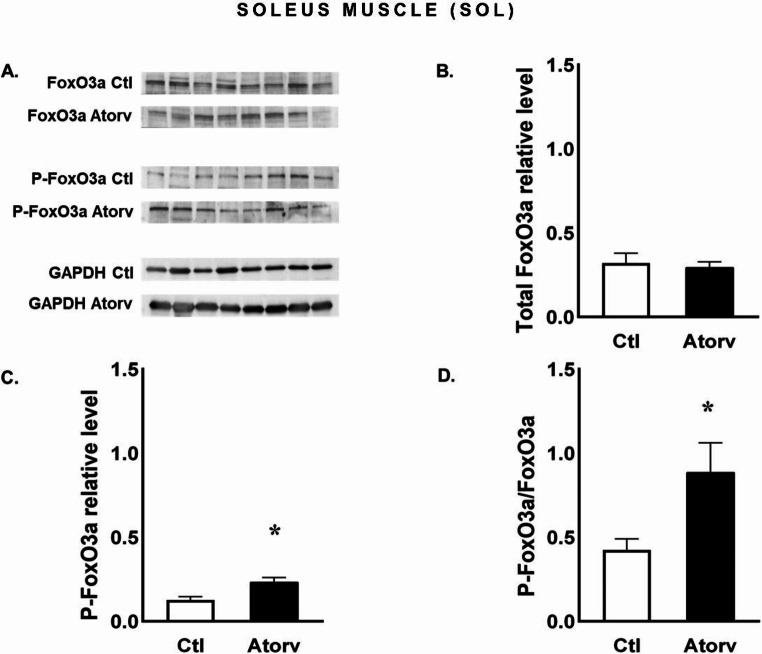
Semi-quantitative analysis of atorvastatin effects on FoxO3a and P-FoxO3a protein levels and the P-FoxO3a/FoxO3a ratio in the SOL muscle performed via Western blotting. **A**: Representative immunoblots for FoxO3a, P-FoxO3a and GAPDH; **B**: Relative FoxO3a protein levels; **C**: Relative P-FoxO3a protein levels; **D**: P-FoxO3a/FoxO3a ratio. *Abbreviations* Ctl, control group; Atorv, atorvastatin-treated group. The data are presented as the means ± SEMs of *n* = 8 animals per group. *p < *0.05*

Atorvastatin treatment significantly increased (*p* < 0.05) FoxO3a phosphorylation in the SOL muscle (Fig. 7C). A significant increase (*p* < 0.05) in the P-FoxO3a/FoxO3a ratio was detected (Fig. 7D), whereas there was no effect (*p* > 0.05) on total FoxO3a levels in the SOL muscle (Fig. 7B).

### Atorvastatin does not affect Akt, rpS6 or FoxO3a in the EDL of rats

**Fig. 8:**
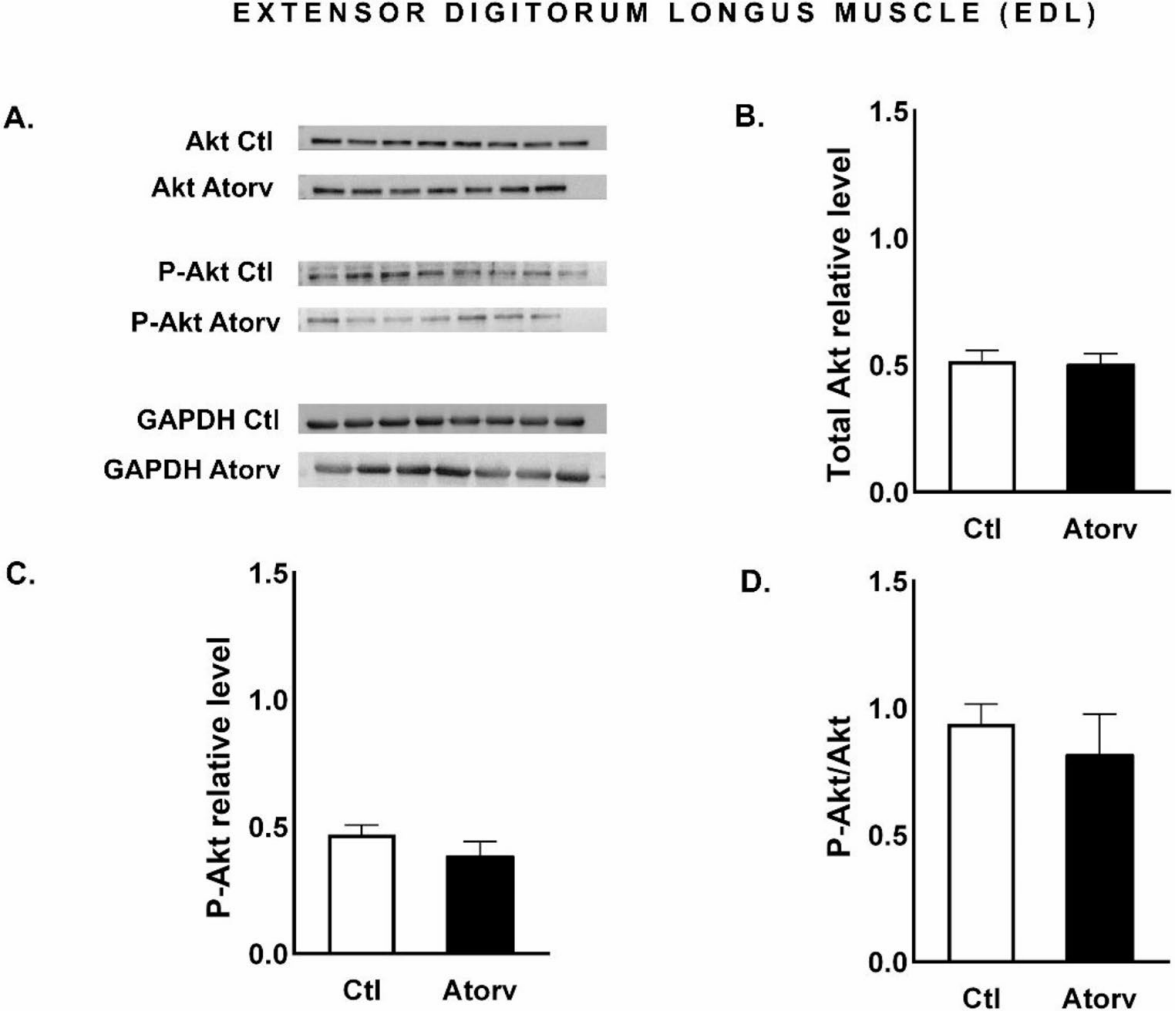
Semi-quantitative analysis of atorvastatin effects on Akt and P-Akt protein levels and the P-Akt/Akt ratio in the EDL muscle performed via Western blotting. **A**: Representative immunoblots for Akt, P-Akt and GAPDH; **B**: Relative Akt protein levels; **C**: Relative P-Akt protein levels; **D**: P-Akt/Akt ratio. *Abbreviations* Ctl, control group; Atorv, atorvastatin-treated group. The data are presented as the means ± SEMs of *n* = 8 animals per Ctl group and *n* = 7 animals per Atrov group

**Fig. 9:**
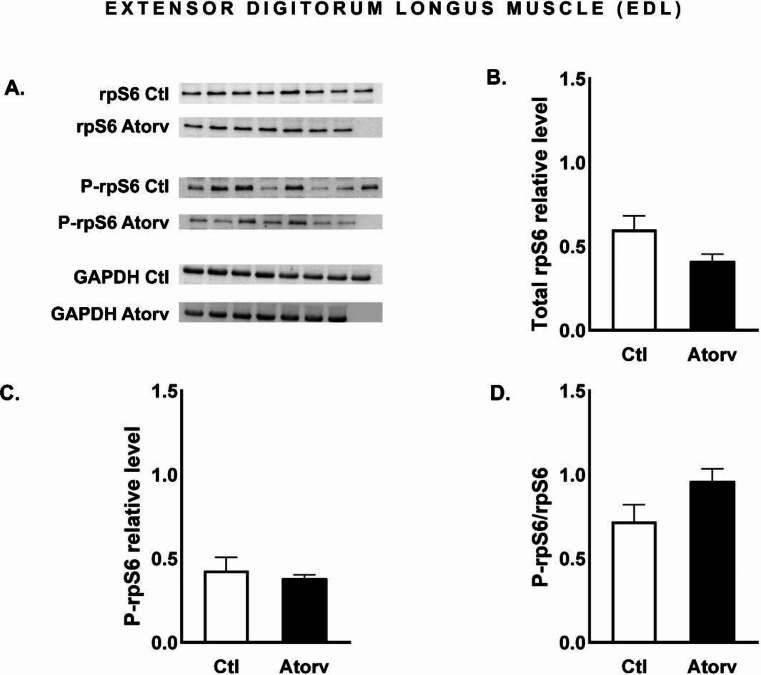
Semi-quantitative analysis of atorvastatin effects on rpS6 and P-rpS6 protein levels and the P-rpS6/rpS6 ratio in the EDL muscle performed via Western blotting. **A**: Representative immunoblots for rpS6, P-rpS6 and GAPDH; **B**: Relative rpS6 protein levels; **C**: Relative P-rpS6 protein levels; **D**: P-rpS6/rpS6 ratio. *Abbreviations* Ctl, control group; Atorv, atorvastatin-treated group. The data are presented as the means ± SEMs of *n* = 8 animals per Ctl group and *n* = 7 animals per Atorv group

**Fig. 10:**
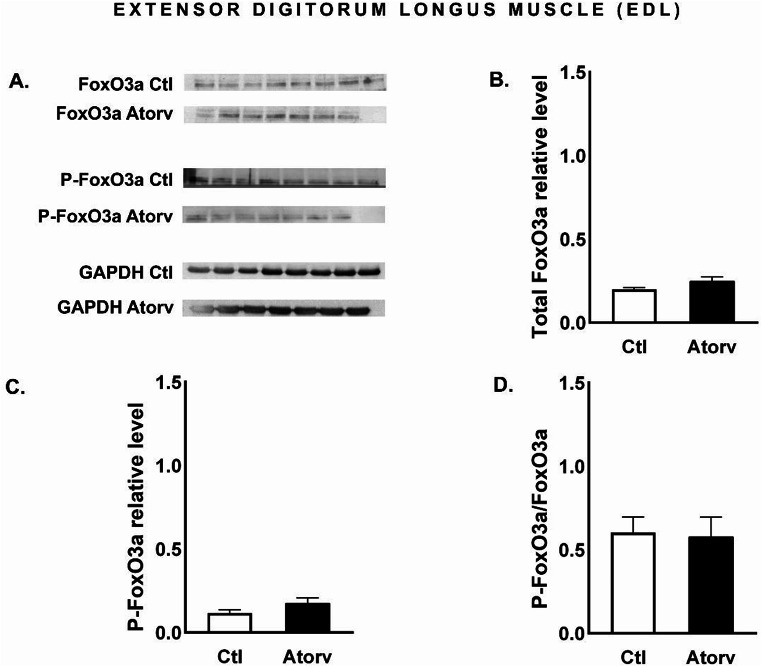
Semi-quantitative analysis of atorvastatin effects on FoxO3a and P-FoxO3a protein levels and the P-FoxO3a/FoxO3a ratio in the EDL muscle performed via Western blotting. **A**: Representative immunoblots for FoxO3a, P-FoxO3a and GAPDH; **B**: Relative FoxO3a protein levels; **C**: Relative P-FoxO3a protein levels; **D**: P-FoxO3a/FoxO3a ratio. *Abbreviations* Ctl, control group; Atorv, atorvastatin-treated group. The data are presented as the means ± SEMs of *n* = 8 animals per Ctl group and *n* = 7 animals per Atorv group

Atorvastatin did not change (*p* > 0.05) the total Akt, total rpS6 or total FoxO3a protein levels. Moreover, the level of phosphorylated proteins remained unchanged (*p* > 0.05), resulting in unaltered P-Akt/Akt, P-rpS6/rpS6 and P-FoxO3a/FoxO3a ratios (Figs. 8, 9 and 10).

### RT-qPCR findings

**Fig. 11:**
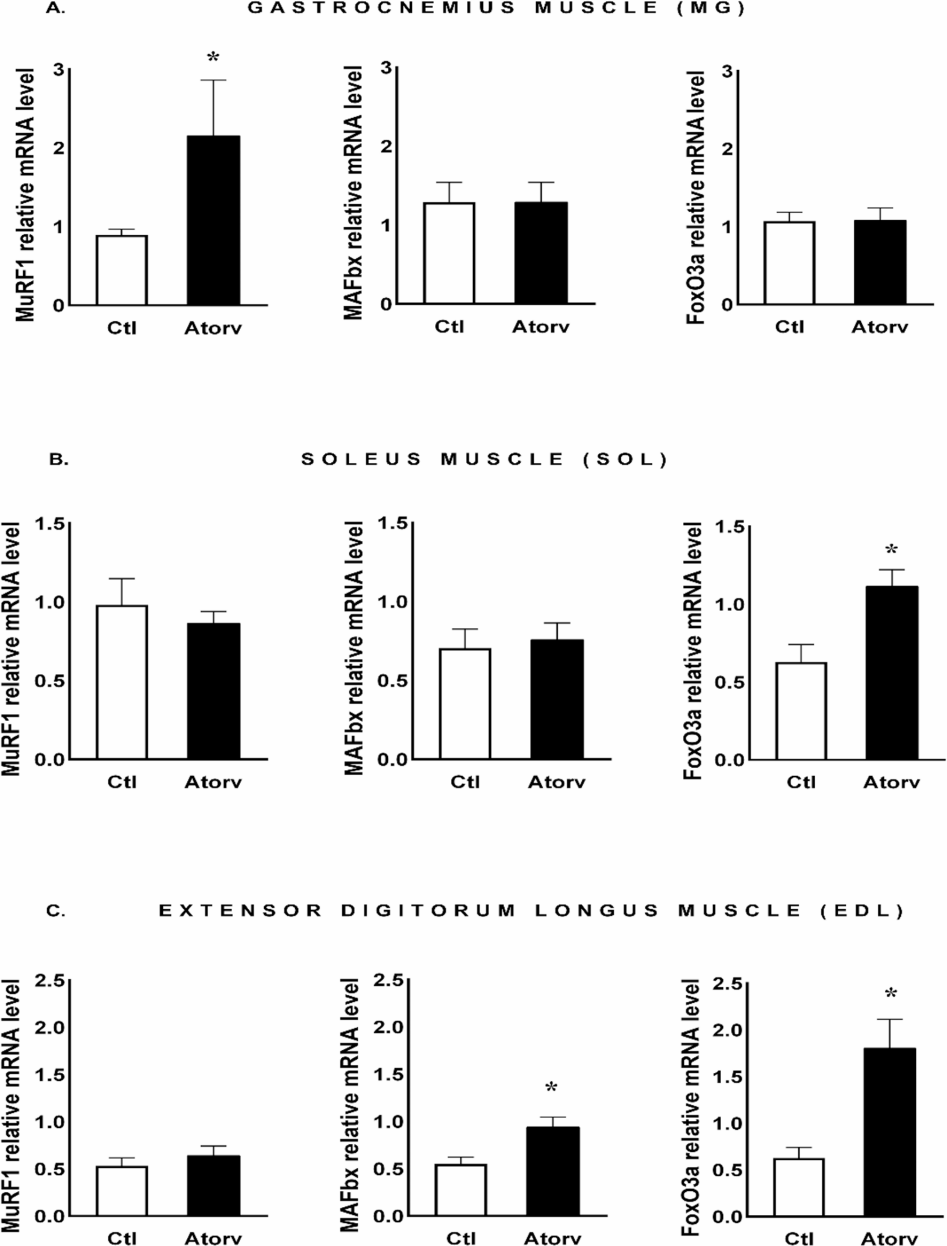
qPCR analysis of atorvastatin effects on MuRF-1, MAFbx and FoxO3a mRNA levels in the **A**: gastrocnemius (MG), **B**: soleus (SOL) and **C**: extensor digitorum longus (EDL) muscles. *Abbreviations* Ctl, control group; Atorv, atorvastatin-treated group. The data are presented as the means ± SEMs of *n* = 8 animals per group for MG and SOL and of *n* = 7 animals per group for EDL. *p < 0.05

To investigate the effect of atorvastatin on the expression of genes involved in skeletal muscle protein degradation, we performed RT‒qPCR assays for MuRF-1, MAFbx and FoxO3a in the MG, SOL and EDL. In the MG, atorvastatin elevated (*p* < 0.05) the level of MuRF-1 mRNA only. The levels of MAFbx and FoxO3a gene expression remained unchanged (*p* > 0.05) (Fig. 11A).

In the SOL muscle, atorvastatin treatment resulted in an increase (*p* < 0.05) in FoxO3a mRNA levels, with no changes (*p* > 0.05) in atrophy-related gene expression, i.e., MuRF-1 and MAFbx (Fig. 11B).

We also found that in the EDL, atorvastatin treatment increased (*p* < 0.05) MAFbx and FoxO3a gene expression but did not affect (*p* > 0.05) MuRF-1 gene expression (Fig. 11C).

### Histopathological findings

To investigate the impact of atorvastatin on skeletal muscle structure, we performed Gomöri trichrome (GT) staining of EDL sections. GT staining revealed no pathological changes in the muscles of the rats in either group. Supplemental Figure S1 shows representative images of the EDL muscle from the Ctl and Atorv groups after GT staining.

## Discussion

The findings of this study demonstrated that 14 days of atorvastatin treatment significantly reduced food intake and body weight in rats. These findings suggest that atorvastatin has an effect on energy balance and metabolism, leading to decreased caloric consumption and subsequent weight loss. However, notably, the reduction in body weight was not accompanied by a decrease in the wet mass of the MG. These findings indicate that the decrease in body weight observed in the atorvastatin-treated rats may be attributed to changes in adipose tissue mass rather than to muscle mass loss. Our results are consistent with the results of others who also reported body weight loss without any skeletal muscle mass loss in statin-treated rats [20].

The diminished food intake and body weight loss after atorvastatin administration may indicate a state of energy deficiency that indirectly affects the PI3K/Akt/mTOR pathway and proteostasis. Under such conditions, the cellular AMP/ATP and ADP/ATP ratios increase. This results in AMPK and FoxO activation, whereas Akt is inhibited [24]. Active AMPK inhibits mTORC1 and thus downregulates protein synthesis by reducing rpS6 and 4E-BP1 phosphorylation [10, 11, 25, 26].

We suspected that the adverse effects of atorvastatin would affect mainly skeletal muscles composed mostly of type II fibres. This finding is based on the results of previous studies showing that statins have greater myotoxic effects on type II fibres than on type I fibres [18, 19]. To investigate the impact of atorvastatin on the PI3K/Akt/mTOR signalling pathway and its downstream effectors in type II fibres, we evaluated total Akt, P-Akt, total rpS6, P-rpS6, total FoxO3a and P-FoxO3a protein levels in the EDL muscle. Surprisingly, atorvastatin did not alter P-Akt levels or the P-Akt/Akt ratio in the EDL muscle. Moreover, we did not observe any changes in the protein levels of downstream effectors, i.e., rpS6 and FoxO3a, or their phosphorylated forms in the Atorv group. These results suggest that short-term atorvastatin administration did not disrupt the Akt pathway in the EDL muscle. These findings are consistent with the results of the histological examination of the EDL muscle. TG staining revealed no difference in the morphologic structure of the EDL muscle between the Ctl and Atorv groups. In addition, EDL histopathological analysis revealed no characteristic markers of mitochondrial damage, which are considered indicators of muscle atrophy.

Furthermore, we did not observe any changes in serum CK activity. Similar results were obtained by Goodman et al. [7], who reported that simvastatin treatment did not alter CK activity. The lack of increase in CK activity does not exclude the presence of alterations in skeletal muscle function. The most frequent type of statin-associated muscle symptom (SAMS) is muscle pain, with no or only mild CK elevation [27]. However, qPCR analysis revealed the upregulation of MAFbx and FoxO3a gene expression. This result is similar to that obtained by Mallinson et al. [28], who reported that short-term simvastatin treatment increased FoxO3a and MAFbx gene expression in type II fibres. The upregulation of gene expression may stimulate skeletal muscle protein degradation.

In the MG, which contains both type I and type II fibres, we did not observe any differences in the relative levels of total Akt and P-Akt, but atorvastatin decreased the P-Akt/Akt ratio. These findings indicate that the inactive form of the Akt protein is predominant and that the PI3K/Akt pathway is likely limited. This result is consistent with those obtained by Seo et al. [29], who demonstrated that atorvastatin decreases the P-Akt/Akt ratio in rat MG. This effect is thought to be associated with PI3K/Akt/mTOR pathway inhibition. The decrease in Akt phosphorylation after atorvastatin administration may be the result of impaired insulin receptor substrate-1 (IRS-1), which plays a crucial role in signal transmission from the insulin receptor (IR) and insulin-like growth factor-1 (IGF-1) to the PI3K/Akt pathway. Statins induce IRS-1 phosphorylation at Ser 636/639 [30] and mediate mTOR-induced inhibition of the Akt signalling pathway via a negative feedback loop [8].

A reduced P-Akt/Akt ratio can contributes to mTORC1 inhibition and inhibition of S6K1 phosphorylation in MG. These changes lead to decreased protein synthesis in skeletal muscle [31]. We found that the atorvastatin-induced decrease in the P-Akt/Akt ratio was accompanied by a marked decrease in P-rpS6 levels in the MG. This result is consistent with others showing that statins reduce P-rpS6 levels in different cell lines, including myoblasts [5, 24, 26]. This finding may suggest that atorvastatin impairs protein synthesis in the MG by affecting signalling pathways such as the mTOR pathway, which is essential for maintaining muscle proteostasis and muscle growth.

The analysis of selected gene expression in the MG revealed elevated MuRF-1 mRNA levels, whereas MAFbx mRNA levels remained unchanged. Recent studies have demonstrated that statins increase the expression of muscle atrophy-related genes [5, 7]. Goodman et al. [7] reported that simvastatin administration at doses of 60 and 80 mg/kg/day for 14 days resulted in significantly increased MuRF-1 and MAFbx gene mRNA expression levels in the MG of rats. However, Bonifacio et al. [5] reported increased mRNA levels of the MAFbx gene only in mice treated with 5 mg/kg/day simvastatin for 21 days. We suspect that a decrease in the P-Akt/Akt ratio, indicating a reduction in the active form of Akt, and an increase in MuRF-1 gene expression may induce protein degradation and disrupt proteostasis in the MG. However, further studies evaluating the effects of atorvastatin on muscle protein degradation/synthesis rates are needed.

To investigate the role of short-term atorvastatin administration on type I muscle fibres, we focused on the SOL muscle. Interestingly, we observed an increase in Akt phosphorylation, i.e., increased P-Akt levels and P-Akt/Akt ratios, which were accompanied by increased P-FoxO3a levels and P-FoxO3a/FoxO3a ratios. These changes indicate a shift towards processes that limit protein degradation, which may be indirectly supported by the lack of changes in the expression of genes encoding E3 ubiquitin ligases, i.e., MuRF-1 and MAFbx, in the SOL muscle. However, we observed no changes in rpS6 phosphorylation. These findings are not consistent with other results that revealed that statins diminish Akt pathway activation [5]. We suspect that the increased Akt phosphorylation and lack of changes in muscle atrophy-related gene expression may be due to the high capacity of type I muscle fibres to maintain metabolic homeostasis, including proteostasis. As previously shown, atorvastatin increases reactive oxygen species (ROS) production in rat skeletal muscles [32]. Oxidative stress can lead to muscle atrophy by stimulating proteolysis and/or inhibiting protein synthesis. It has been demonstrated that type I muscle fibres exhibit greater oxidative capacity and greater efficiency in inactivating ROS than type II muscle fibres do [33, 34].

Notably, this study has several limitations. This study focused on the evaluation of total and phosphorylated protein levels in the PI3K/Akt/mTOR signalling pathway and atrophy-related gene expression, but it did not include a direct evaluation of the protein synthesis/degradation rate.

## Conclusions

We demonstrated that in a model that was not expected to induce overt muscle damage, atorvastatin decreased the P-Akt/Akt ratio indicating that the inactive form of the Akt protein predominated. Consistent with the decrease in Akt activation, rpS6 phosphorylation decreased. In contrast, in the SOL muscle, atorvastatin increased the P-Akt/Akt ratio, indicating the activation of Akt. Consistent with the findings of Akt activation, FoxO3a phosphorylation and the P-FoxO3a/FoxO3a ratio increased, implying that FoxO3 inactivation occurred. Moreover, in the SOL muscle, atorvastatin did not affect the expression of atrophy-related genes. These findings indicate that atorvastatin has no adverse effect on the Akt pathway in the SOL muscle. Our results showed that the effects of atorvastatin on the Akt signalling pathway and atrophy-related gene expression depend on muscle type.

## Electronic supplementary material

Below is the link to the electronic supplementary material.

Supplementary Material 1

## Data Availability

Data that support the findings of this study have been deposited in the RODBUK Cracow Open Research Data Repository available at: 10.57903/UJ/RQIULT.

## Abbreviations

4E-BP1: Eukaryotic initiation factor 4E-binding protein 1
Akt: Protein kinase B
CK: Creatine kinase
EDL: Extensor digitorum longus
FoxO3a: Forkhead box transcription factor
GT: Gomöri trichrome
HMG-CoA: 3-Hydroxy-3-Methyl-Glutaryl-CoA
MAFbx: Muscle atrophy F-box
MG: Gastrocnemius muscle
MuRF-1: Muscle RING finger-1
mTORC: Mammalian target of rapamycin kinase
qPCR: Quantitative polymerase chain reaction
rpS6: Ribosomal protein S6
S6K1: Ribosomal protein S6 kinase
SAMS: Statins-associated muscle symptoms
SOL: Soleus muscle
UPS: Ubiquitin-proteasome system

## References

[1] Ward NC, Watts GF, Eckel RH (2019) Statin toxicity. Circ Res 124:328–350. 10.1161/CIRCRESAHA.118.312782 30653440 10.1161/CIRCRESAHA.118.312782

[2] Matyori A, Brown CP, Ali A, Sherbeny F (2023) Statins utilization trends and expenditures in the U.S. before and after the implementation of the 2013 ACC/AHA guidelines. Saudi Pharm J 31:795–800. 10.1016/j.jsps.2023.04.002 37228328 10.1016/j.jsps.2023.04.002 PMC10203693

[3] Hoste E, Haufroid V, Deldicque L, Balligand JL, Elens L (2024) Atorvastatin-associated myotoxicity: a toxicokinetic review of pharmacogenetic associations to evaluate the feasibility of precision pharmacotherapy. Clin Biochem 124:110707. 10.1016/j.clinbiochem.2024.110707 38182100 10.1016/j.clinbiochem.2024.110707

[4] Mueller AM, Liakoni E, Schneider C, Burkard T, Jick SS, Krähenbühl S, Meier CR, Spoendlin J (2021) The risk of muscular events among new users of hydrophilic and lipophilic statins: an Observational Cohort Study. J Gen Intern Med 36(9):2639–2647. 10.1007/s11606-021-06651-6 33751411 10.1007/s11606-021-06651-6 PMC8390626

[5] Bonifacio A, Sanvee GM, Bouitbir J, Krähenbühl S (2015) The AKT/mTOR signaling pathway plays a key role in statin-induced myotoxicity. Biochim Biophys Acta 1853:1841–1849. 10.1016/j.bbamcr.2015.04.010 25913013 10.1016/j.bbamcr.2015.04.010

[6] Camerino GM, Tarantino N, Canfora I, De Bellis M, Musumeci O, Pierno S (2021) Statin-Induced Myopathy: translational studies from preclinical to clinical evidence. Int J Mol Sci 22(4):2070. 10.3390/ijms22042070 33669797 10.3390/ijms22042070 PMC7921957

[7] Goodman CA, Pol D, Zacharewicz E, Lee-Young RS, Snow RJ, Russell AP, McConell GK (2015) Statin-induced increases in atrophy gene expression occur independently of changes in PGC1α protein and mitochondrial content. PLoS ONE 10:e0128398. 10.1371/journal.pone.0128398 26020641 10.1371/journal.pone.0128398 PMC4447258

[8] Tzatsos A, Kandror KV (2006) Nutrients suppress phosphatidylinositol 3-kinase/Akt signaling via raptor-dependent mTOR-mediated insulin receptor substrate 1 phosphorylation. Mol Cell Biol 26:63–76. 10.1128/MCB.26.1.63-76.2006 16354680 10.1128/MCB.26.1.63-76.2006 PMC1317643

[9] Hermida MA, Dinesh Kumar J, Leslie NR (2017) GSK3 and its interactions with the PI3K/AKT/mTOR signalling network. Adv Biol Regul 65:5–15. 10.1016/j.jbior.2017.06.003 28712664 10.1016/j.jbior.2017.06.003

[10] Fenton TR, Gout IT (2011) Functions and regulation of the 70 kDa ribosomal S6 kinases. Int J Biochem Cell Biol 43:47–59. 10.1016/j.biocel.2010.09.018 20932932 10.1016/j.biocel.2010.09.018

[11] Memmott RM, Dennis PA (2009) Akt-dependent and independent mechanisms of mTOR regulation in cancer. Cell Signal 21:656–664. 10.1016/j.cellsig.2009.01.004 19166931 10.1016/j.cellsig.2009.01.004 PMC2650010

[12] Kang SH, Lee HA, Kim M, Lee E, Sohn UD, Kim I (2017) Forkhead box O3 plays a role in skeletal muscle atrophy through expression of E3 ubiquitin ligases MuRF-1 and atrogin-1 in Cushing’s syndrome. Am J Physiol Endocrinol Metab. 10.1152/ajpendo.00389.2016 10.1152/ajpendo.00389.2016 28246104

[13] Bodine SC, Baehr LM (2014) Skeletal muscle atrophy and the E3 ubiquitin ligases MuRF1 and MAFbx/atrogin-1. Am J Physiol Endocrinol Metab 307(6):E469–E484. 10.1152/ajpendo.00204.2014 25096180 10.1152/ajpendo.00204.2014 PMC4166716

[14] Chen K, Gao P, Li Z, Dai A, Yang M, Chen S, Su J, Deng Z, Li L (2022) Forkhead Box O signaling pathway in skeletal muscle atrophy. Am J Pathol 192(12):1648–1657. 10.1016/j.ajpath.2022.09.003 36174679 10.1016/j.ajpath.2022.09.003

[15] Soukup T, Zacharová G, Smerdu V (2002) Fibre type composition of soleus and extensor digitorum longus muscles in normal female inbred Lewis rats. Acta Histochem 104(4):399–405. 10.1078/0065-1281-00660 12553710 10.1078/0065-1281-00660

[16] Santocildes G, Merino M, Fabiani F, Pagès T, Marotta M, Viscor G, Torrella JR (2022) Histomorphological and functional contralateral symmetry in the gastrocnemius muscles of the laboratory rat. J Anat 241(3):692–701. 10.1111/joa.13674 35437750 10.1111/joa.13674 PMC9358741

[17] Armstrong RB, Phelps RO (1984) Muscle fiber type composition of the rat hindlimb. Am J Anat 171(3):259–272. 10.1002/aja.1001710303 6517030 10.1002/aja.1001710303

[18] Westwood FR, Scott RC, Marsden AM, Bigley A, Randall K (2008) Rosuvastatin: characterization of induced myopathy in the rat. Toxicol Pathol 36:345–352. 10.1177/0192623307311412 18362199 10.1177/0192623307311412

[19] Westwood FR, Bigley A, Randall K, Marsden AM, Scott RC (2005) Statin-induced muscle necrosis in the rat: distribution, development, and fibre selectivity. Toxicol Pathol 33:246–257. 10.1080/01926230590908213 15902968 10.1080/01926230590908213

[20] Simsek Ozek N, Bal IB, Sara Y, Onur R, Severcan F (2014) Structural and functional characterization of simvastatin-induced myotoxicity in different skeletal muscles. Biochim Biophys Acta 1840:406–415. 10.1016/j.bbagen.2013.09.010 24045089 10.1016/j.bbagen.2013.09.010

[21] Elshama SS, El-Kenawy AE-M, Osman H-EH (2016) Curcumin improves atorvastatin-induced myotoxicity in rats: histopathological and biochemical evidence. Int J Immunopathol Pharmacol 29:742–752. 10.1177/0394632016656186 27507589 10.1177/0394632016656186 PMC5806835

[22] Gawedzka A, Drag J, Knapik-Czajka M, Belczyk M, Szafran A, Szlachta I (2023) Skeletal muscle type-dependent effect of atorvastatin on FoxO3a and akt in hypercholesterolemic rats. Acta Pol Pharm - Drug Res 80(5):841–847. 10.32383/appdr/174241

[23] Knapik-Czajka M, Gawędzka A, Jurczyk M, Drąg J, Belczyk M, Aleksanrovych V, Gil A, Gil K (2022) The influence of 5-fluorouracil on the α-ketoglutarate dehydrogenase complex in rat’s cardiac muscle - a preliminary study. Folia Med Cracov 62:27–35. 10.24425/fmc.2022.141705 36256893 10.24425/fmc.2022.141705

[24] Hardie DG, Hawley SA (2001) AMP-activated protein kinase: the energy charge hypothesis revisited. BioEssays 23:1112–1119. 10.1002/bies.10009 11746230 10.1002/bies.10009

[25] Castets P, Rion N, Théodore M, Falcetta D, Lin S, Reischl M, Wild F, Guérard L, Eickhorst C, Brockhoff M, Guridi M, Ibebunjo C, Cruz J, Sinnreich M, Rudolf R, Glass DJ, Rüegg MA (2019) mTORC1 and PKB/Akt control the muscle response to denervation by regulating autophagy and HDAC4. Nat Commun 10(1):3187. 10.1038/s41467-019-11227-4 31320633 10.1038/s41467-019-11227-4 PMC6639401

[26] Vadlakonda L, Dash A, Pasupuleti M, Anil Kumar K, Reddanna P (2013) The paradox of Akt-mTOR interactions. Front Oncol 3:165. 10.3389/fonc.2013.00165 23802099 10.3389/fonc.2013.00165 PMC3687210

[27] Phillips PS, Haas RH, Bannykh S, Hathaway S, Gray NL, Kimura BJ, Vladutiu GD, England JD (2002) Statin-associated myopathy with normal creatine kinase levels. Ann Intern Med 137:581–585. 10.7326/0003-4819-137-7-200210010-00009 12353945 10.7326/0003-4819-137-7-200210010-00009

[28] Mallinson JE, Constantin-Teodosiu D, Sidaway J, Westwood FR, Greenhaff PL (2009) Blunted Akt/FOXO signalling and activation of genes controlling atrophy and fuel use in statin myopathy. J Physiol 587(1):219–230. 10.1113/jphysiol.2008.164699 19001041 10.1113/jphysiol.2008.164699 PMC2670035

[29] Seo DY, Heo JW, No MH, Yoo SZ, Ko JR, Park DH, Kang JH, Kim CJ, Jung SJ, Han J, Kwak HB (2020) Exercise training protects against atorvastatin-induced skeletal muscle dysfunction and mitochondrial dysfunction in the skeletal muscle of rats. J Clin Med 9:2292. 10.3390/jcm9072292 32707695 10.3390/jcm9072292 PMC7408828

[30] Roudier E, Mistafa O, Stenius U (2006) Statins induce mammalian target of rapamycin (mTOR)-mediated inhibition of akt signaling and sensitize p53-deficient cells to cytostatic drugs. Mol Cancer Ther 5:2706–2715. 10.1158/1535-7163.MCT-06-0352 17121917 10.1158/1535-7163.MCT-06-0352

[31] Ruvinsky I, Meyuhas O (2006) Ribosomal protein S6 phosphorylation: from protein synthesis to cell size. Trends Biochem Sci 31(6):342–348. 10.1016/j.tibs.2006.04.003 16679021 10.1016/j.tibs.2006.04.003

[32] Bouitbir J, Charles AL, Rasseneur L, Dufour S, Piquard F, Geny B, Zoll J (2011) Atorvastatin treatment reduces exercise capacities in rats: involvement of mitochondrial impairments and oxidative stress. J Appl Physiol (1985) 111(5):1477–1483. 10.1152/japplphysiol.00107.2011 21852406 10.1152/japplphysiol.00107.2011

[33] Bouitbir J, Singh F, Charles AL, Schlagowski AI, Bonifacio A, Echaniz-Laguna A, Geny B, Krähenbühl S, Zoll J (2016) Statins trigger mitochondrial reactive Oxygen species-Induced apoptosis in glycolytic skeletal muscle. Antioxid Redox Signal 24(2):84–98. 10.1089/ars.2014.6190 26414931 10.1089/ars.2014.6190

[34] Bouitbir J, Sanvee GM, Panajatovic MV, Singh F, Krähenbühl S (2020) Mechanisms of statin-associated skeletal muscle-associated symptoms. Pharmacol Res 154:104201. 10.1016/j.phrs.2019.03.010 30877064 10.1016/j.phrs.2019.03.010

