# Supplementary material for: Skeletal muscle fibre type-dependent effects of atorvastatin on the PI3K/Akt/mTOR signalling pathway and atrophy-related genes in rats
Source: Mol Biol Rep. 2024 Oct 17;51(1):1062. doi: 10.1007/s11033-024-10005-w (PMC11486814; doi:10.1007/s11033-024-10005-w)
Supplement: Supplementary file 1 — Supplementary Material 1 [file 11033_2024_10005_MOESM1_ESM.docx]

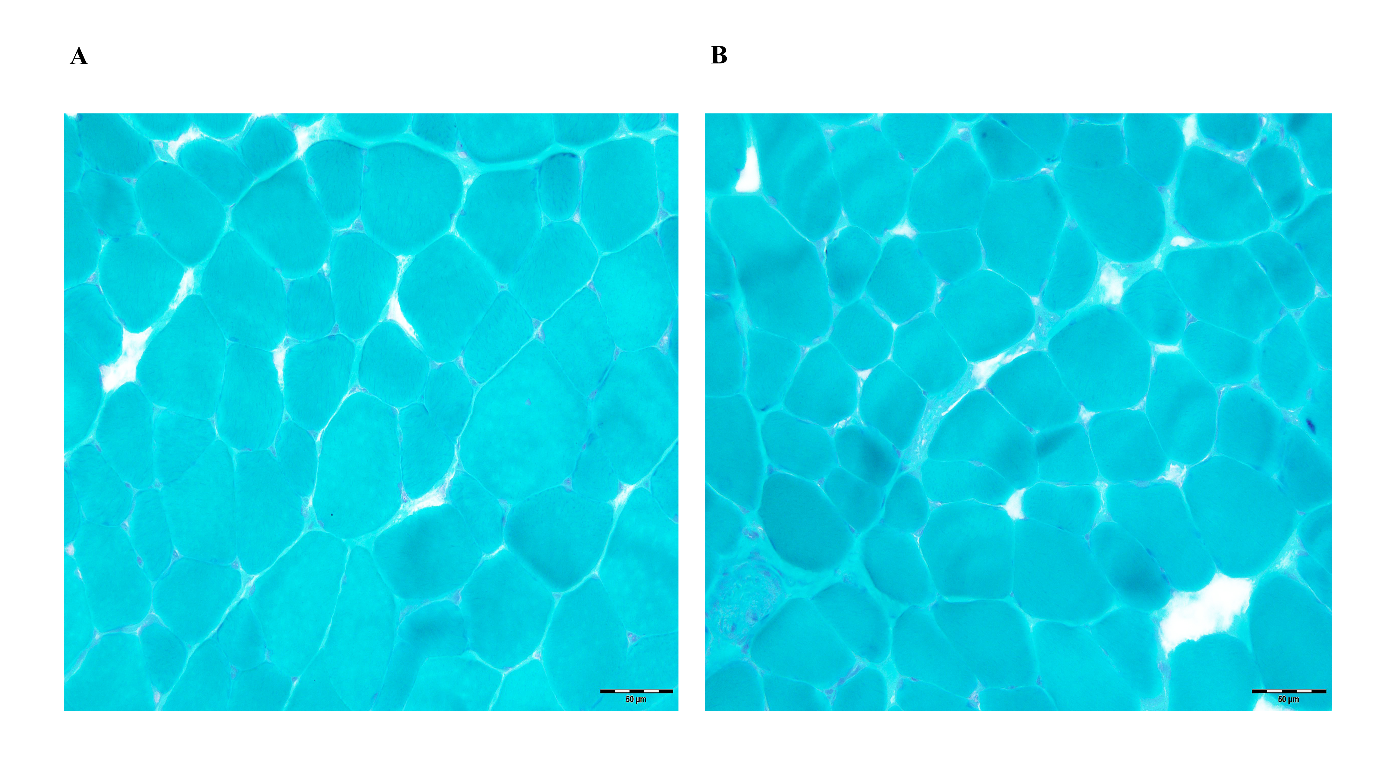


Fig. S1 Histological analysis of the EDL. Representative histological images of the EDL muscle stained via the Gomöri trichrome method. A: Ctl, control group; B: Atorv, atorvastatin-treated group.
